# Proteome changes in the skin of the grape cultivar Barbera among different stages of ripening

**DOI:** 10.1186/1471-2164-9-378

**Published:** 2008-08-08

**Authors:** Alfredo S Negri, Bhakti Prinsi, Mara Rossoni, Osvaldo Failla, Attilio Scienza, Maurizio Cocucci, Luca Espen

**Affiliations:** 1 Dipartimento di Produzione Vegetale, University of Milan, via Celoria 2, 20133 Milano, Italy

## Abstract

**Background:**

Grape ripening represents the third phase of the double sigmoidal curve of berry development and is characterized by deep changes in the organoleptic characteristics. In this process, the skin plays a central role in the synthesis of many compounds of interest (*e.g*. anthocyanins and aroma volatiles) and represents a fundamental protective barrier against damage by physical injuries and pathogen attacks. In order to improve the knowledge on the role of this tissue during ripening, changes in the protein expression in the skin of the red cultivar Barbera at five different stages from *véraison *to full maturation were studied by performing a comparative 2-DE analysis.

**Results:**

The proteomic analysis revealed that 80 spots were differentially expressed throughout berry ripening. Applying a two-way hierarchical clustering analysis to these variations, a clear difference between the first two samplings (up to 14 days after *véraison*) and the following three (from 28 to 49 days after *véraison*) emerged, thus suggesting that the most relevant changes in protein expression occurred in the first weeks of ripening. By means of LC-ESI-MS/MS analysis, 69 proteins were characterized. Many of these variations were related to proteins involved in responses to stress (38%), glycolysis and gluconeogenesis (13%), C-compounds and carbohydrate metabolism (13%) and amino acid metabolism (10%).

**Conclusion:**

These results give new insights to the skin proteome evolution during ripening, thus underlining some interesting traits of this tissue. In this view, we observed the ripening-related induction of many enzymes involved in primary metabolism, including those of the last five steps of the glycolytic pathway, which had been described as down-regulated in previous studies performed on whole fruit. Moreover, these data emphasize the relevance of this tissue as a physical barrier exerting an important part in berry protection. In fact, the level of many proteins involved in (a)biotic stress responses remarkably changed through the five stages taken into consideration, thus suggesting that their expression may be developmentally regulated.

## Background

Grape berry is a typical true fruit originating from the ovary and is formed by skin, flesh, seeds and a complete vascular system. They all have specific properties that are directly linked to their particular physiological roles during berry development and seed dispersal.

The growth of this non-climacteric fruit is summarized by the well known double-sigmoidal curve and is divided into an initial and rapid growth, a subsequent lag phase and a second period of growth corresponding to berry ripening [1,2]. During the first phase, embryo formation takes place in the seeds and the berry enlarges through frequent cell divisions, accompanied by the accumulation of many solutes, such as malic acid, tartaric acid and tannins [3,4]. The lag phase is characterized by the lack of any changes in berry weight and volume and its end coincides with the onset of ripening. This stage, which is referred to the French word *véraison*, is detectable in red cultivars where the change in skin colour takes place due to the start of anthocyanins synthesis. It is important to observe that at this time phloem unloading shifts to an apoplasmic pathway that is accompanied by a parallel change of the role of xylem in the water budgets [5-7]. Furthermore, ripening is characterized by profound changes in berry composition. The concentrations of some metabolites, among which malic acid is the most important, decrease while the levels of other molecules, such as glucose, fructose, volatile aroma compounds and anthocyanins (in red cultivars), greatly increase [4,8-10]. Moreover, berries start to soften at *véraison *and this event is mainly linked to significant changes in the cell wall composition [11-14].

In all growth phases, the very active metabolism of the skin deeply influences the final characteristics of the grape berry. This tissue, which is formed by a single layer of clear epidermal cells and a few hypodermal layers beneath the epidermis, is in fact the site of the synthesis of anthocyanins and aroma compounds [4,8,10,15] and also represents a fundamental protective barrier against damage by physical injuries and pathogen attacks [16]. The composition of this tissue depends on both the particular genetic background of the cultivar and the environmental conditions. These factors play a central role in influencing colour, aroma and other organoleptic properties of wine [4,17-21].

The impact of gene and protein expression patterns in determining the specificity of the skin in comparison to the other berry tissues is a crucial aspect that must be considered. In this view, two recent studies of the mRNA expression profiles in isolated skins have been published using oligonucleotide or cDNA microarrays [17,22]. Waters and co-workers provided a first description on the main events characterizing the shift in gene expression in this tissue around *véraison *[22]. On the other hand, Grimplet and co-workers compared the mRNA expression profiles of the three major tissues of the berry (skin, pulp and seeds) at maturity. The results of this analysis highlighted that the skin transcriptome presented the most distant fingerprint from the global set, since the categories related to housekeeping processes (*i.e*. protein fate, cell cycle and DNA processing) were under-represented while those related to secondary, amino acid and lipid metabolism were highly expressed, if compared to pulp and seeds [17].

The widening of genomic information obtained in the last few years has also paved the way to the study of protein expression. Recently, some proteomic studies have been performed on grape berry. A 2-DE analysis of the mesocarp profile conducted by Sarry and co-workers [23] allowed the identification of 67 proteins using MALDI-MS, thus providing clues to the sugar and organic acid metabolism in ripe berry pulp. More recently, the first analysis of the skin proteome has been performed by comparing, two by two, three different ripening stages in Cabernet Sauvignon berries [24]. This paper mainly reports differences in the expression of pathogenesis-related proteins and of some enzymes involved in anthocyanin biosynthesis. Giribaldi and co-workers [25], on the other hand, focused their attention on the proteome of whole berries of cv. Nebbiolo during a longer period of time, ranging from one month after flowering to complete ripening. These studies provided a first profile of grape proteomes, also describing some dynamic changes taking place in growing berries, although further efforts are still necessary in order to unravel the physiological events that characterize the grape berry ripening and the specific roles of the different tissues at the protein level.

A crucial step in a 2-DE analysis is the procedure adopted for protein extraction. As with many other fruits, grape is a recalcitrant plant material because of the high concentration of interfering compounds such as phenolics, terpenes, organic acids, ions, carbohydrates and proteolytic and oxidative enzymes [26-31]. This aspect is particularly onerous for investigations of the skin, where some of these compounds are present at very high concentrations. For this tissue, the phenol extraction method followed by ammonium acetate in methanol precipitation appears to be the most appropriate protocol up to now [24,32].

In order to obtain further information on protein expression changes in the skin during berry ripening, a comparative 2-DE analysis was performed on a time-course experimental design made up of five different stages from *véraison *to full ripening of Barbera, a widely cultivated red variety typical of northern Italy. In order to associate the proteome changes to the events characterizing the ripening process, some biochemical parameters were also measured. In this study, it was reported that 80 spots significantly changed their relative volumes among the different stages. Sixty-nine of them were identified by LC-ESI-MS/MS and the corresponding proteins were classified on the basis of their putative functions. Some of these proteins were associated with glycolysis and other carbohydrate pathways of the primary metabolism and were found to increase in the skin tissue during ripening.

## Results and discussion

### 2-DE and image analysis

2-DE analysis was performed on five consecutive stages of ripening that, as described in the Methods section, were also defined through the determination of some physiological parameters.

**Figure 1:**
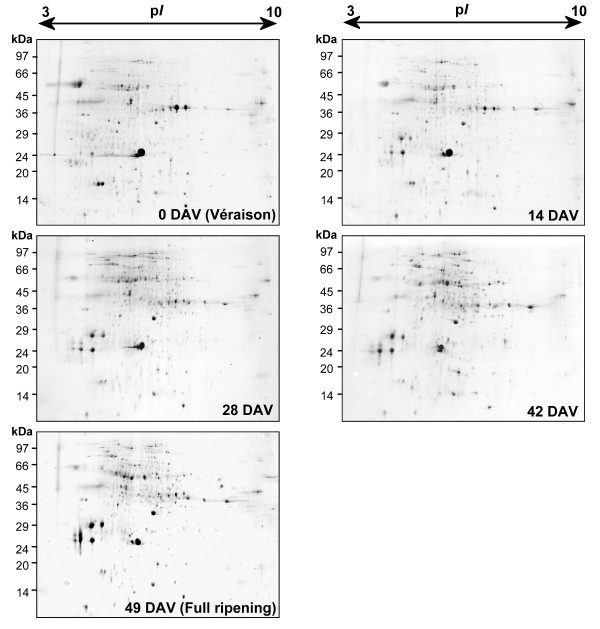
**2-DE maps of five stages through the ripening of Barbera**. 2-DE maps of five different ripening stages from *véraison *until full ripeness of cultivar Barbera berry skins. The *véraison *stage (0 DAV) was considered as the moment when 50% of the berries started to change colour. Proteins (200 μg) were separated by IEF at pH 3–10, followed by 12.5% SDS PAGE and visualized by cCBB-staining.

Proteins were extracted from the berry skin samples of cultivar Barbera previously washed in acetone through a protocol which made use of phenol followed by precipitation in ammonium acetate in methanol, which was previously indicated to be appropriate for this recalcitrant tissue [24]. 2-DE gels are shown in Fig. 1. The average number of detected spots was about 850 for each stage and did not vary significantly among the five different conditions. To ascertain the quantitative changes in the proteomic maps, their relative spot volumes (*%Vol*) were evaluated by software-assisted analysis. The ANOVA test (*p *< 0.01), coupled with a threshold of two-fold change in level, revealed 80 spots as being differentially expressed throughout berry ripening.

### Hierarchical clustering analysis

**Figure 2:**
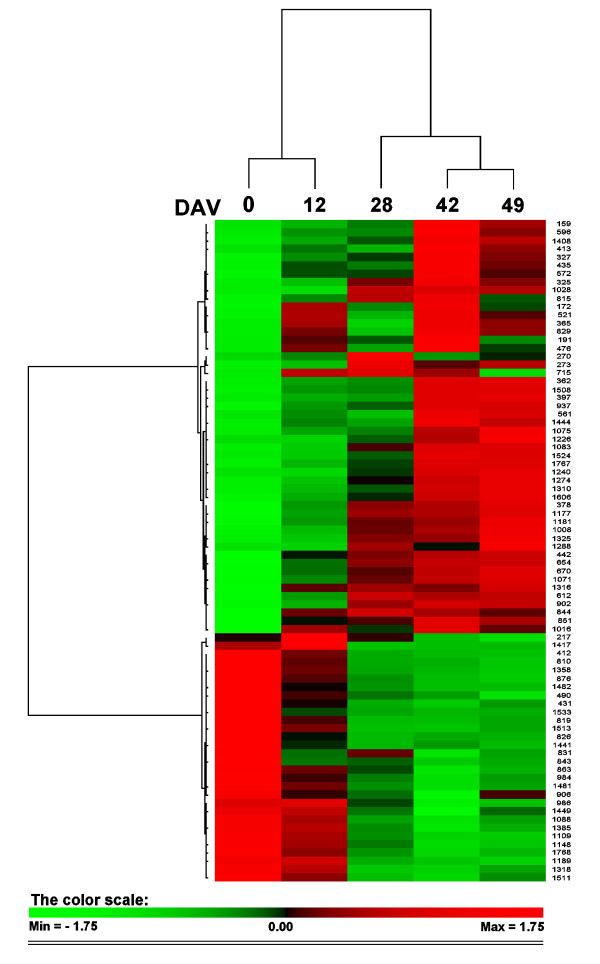
**Clustering analysis of the spots that resulted to change their relative volumes during ripening**. Two-way hierarchical clustering analysis of the 80 spots that showed at least a two-fold change in the relative spot volumes (ANOVA, *p *< 0.01) in the five different ripening stages of grape berry skins of cultivar Barbera. The *véraison *stage (0 DAV) was considered as the moment when 50% of the berries started to change colour. The clustering analysis was performed with PermutMatrix graphical interface after Z-score normalization of the averages of relative spot values (n = 6). Pearson's distance and Ward's algorithm were used for the analysis. Each coloured cell represents the average of the relative spot value, according to the colour scale at the bottom of the figure.

The differentially expressed spots were subjected to two-way hierarchical clustering analysis using the PermutMatrix software (Figure 2). Looking at the clustering of columns, which mirrors the distances among the different stages of berry ripening, it is evident that the bunch order reflects the sequential succession of samples, while there is a clear difference between the first two samplings and the following three. These results suggested that the most important changes in protein expression took place between the second and the third stage. Nevertheless, this behaviour appeared different from the data emerging from oligo/microarrays studies in which the most dramatic changes in the transcriptome were found immediately after *véraison *and appeared well correlated with the start of the ripening process [22,33]. The fact that most of the observed changes in this proteomic analysis did not refer to *véraison*, but to a period between 14 and 28 days after *véraison *(DAV), may reflect peculiar features of cv. Barbera that is characterized by a longer period of ripening, compared to the cultivar Shiraz which was used in the works cited above. Anyway, it is important to underline that comparisons among studies using different genotypes need to be evaluated with extreme caution. In addition, when dealing with in-field grown plants, the relevance of environmental factors should not be excluded since they affect the gene and/or protein expression [18].

As for the row clustering, two main trends were observed: one related to proteins whose levels increase during maturation, the other describing those declining as the berry ripens. Most of the spots belonged to the first class (62.5%), agreeing with the observation that the number of genes whose expression is switched on during ripening is far greater than the amount of genes switched off [22]. The clustering analysis also indicated that the trends of expression relative to the last two ripening stages were closely grouped, suggesting that no evident expression changes took place in that period.

### Protein identification and functional distribution

**Figure 3:**
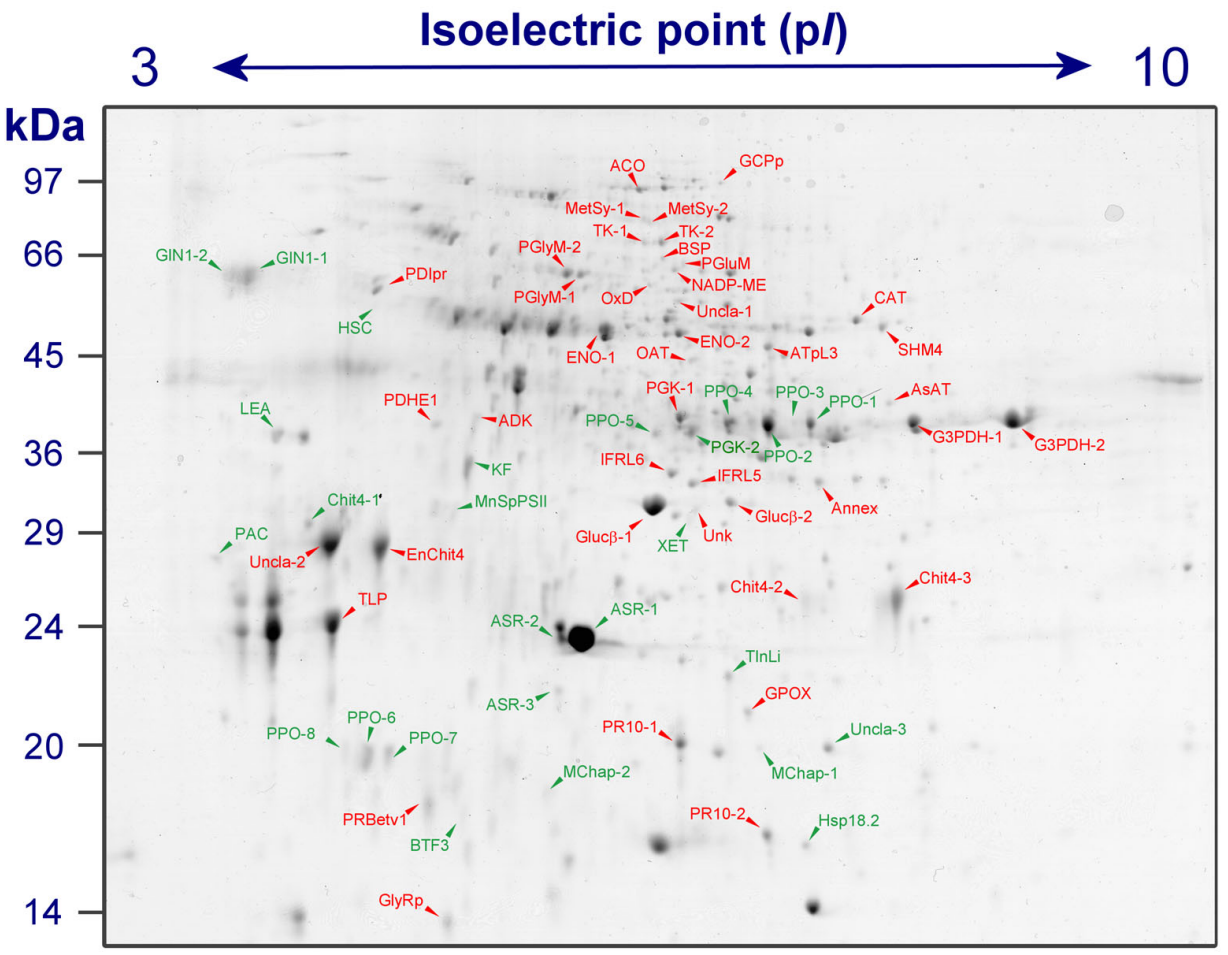
**Protein profiles of identified proteins**. Identified proteins are indicated in a 2-DE gel representative of the fifth ripening stage with spot name abbreviation corresponding to those in Table 1, Figure 6 and 7. Spots showing an increased or a decreased expression during ripening are indicated in red and in green, respectively.

**Figure 4:**
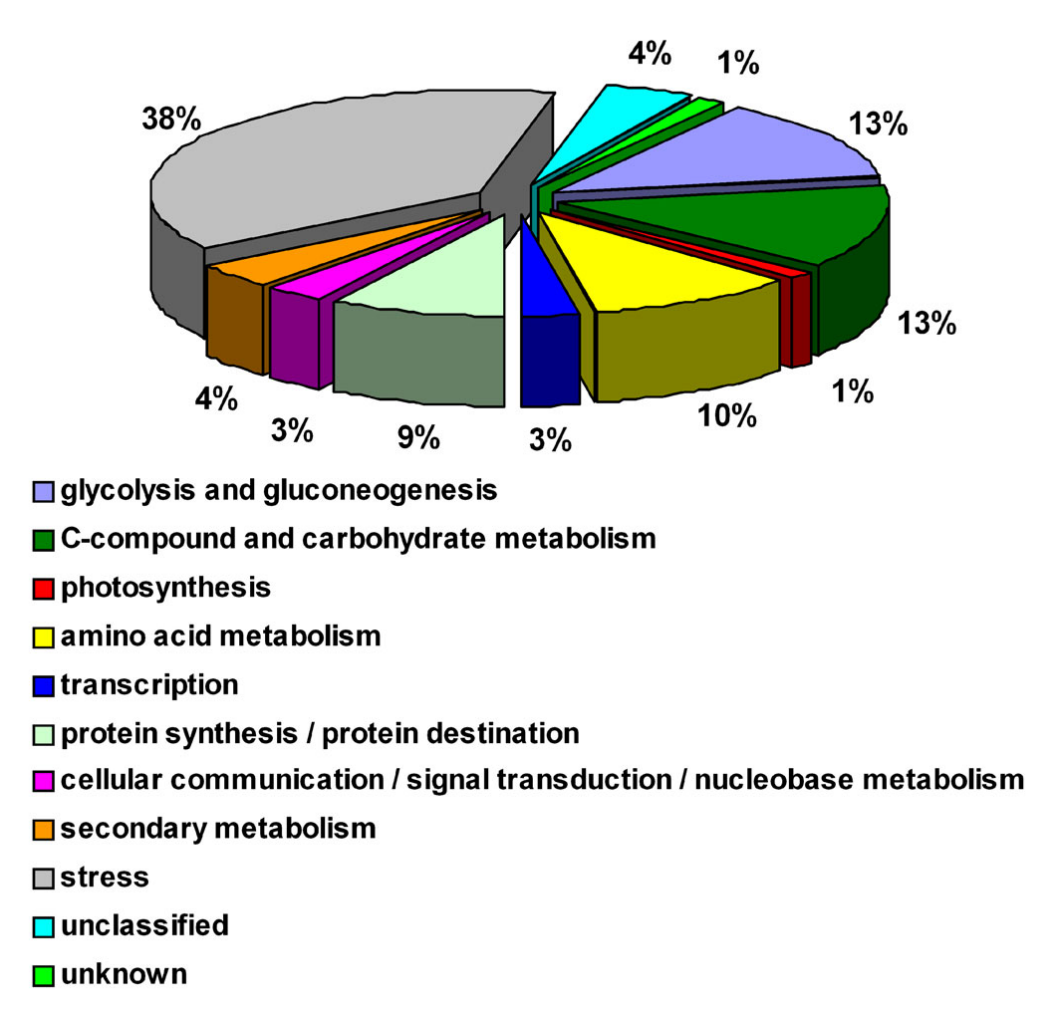
**Functional categories distribution of the identified proteins**. Functional distribution of the identified proteins (Table 1) according to the annotation in the MIPS FunCat.

Among the 80 differentially expressed spots analyzed by LC-ESI-MS/MS, 69 were identified, listed in Table 1 and shown in Figure 3 which is referred to a gel of the fifth stage. The functional distribution of the identified proteins was performed according to MIPS FunCat annotation and is shown in Figure 4.

**Table 1:** List of spots identified by LC-ESI-MS/MS and bioinformatic analysis. Proteins were classified according to MIPS FunCat. Additional data about mass spectrometry are reported in the additional file 1.

Spot ID	Accession number	Protein description	Name abbreviation	M_r_^***a***^	M_r_^***b***^	p*I*^***a***^	p*I*^***b***^	a.a. cov.^***c ***^(%)
**Glycolysis and gluconeogenesis**								
365	Q9ZSQ4	Cytoplasmic phosphoglucomutase	**PGluM**	68.17	63.12	6.16	5.49	9.6
397	Q42908	2,3-bisphosphoglycerate-independent phosphoglycerate mutase	**PGlyM-1**	63.34	61.18	5.83	5.39	5.7
561	P42896	Enolase	**ENO-1**	52.48	47.91	5.89	5.56	35.5
596	P42896	Enolase	**ENO-2**	52.04	47.91	6.14	5.56	31.5
829	CAN81988	Phosphoglycerate kinase^**(*d*)**^	**PGK-1**	40.66	42.42	6.15	6.29	27.9
863	CAN81988	Phosphoglycerate kinase^**(*d*)**^	**PGK-2**	39.06	42.42	6.21	6.29	37.1
902	ABC75834	Glyceraldehyde-3-phosphate dehydrogenase	**G3PDH-1**	37.48	36.76	7.48	6.72	25.1
937	P26518	Glyceraldehyde-3-phosphate dehydrogenase	**G3PDH-2**	36.77	36.98	7.94	7.09	22.0
1767	Q42908	2,3-bisphosphoglycerate-independent phosphoglycerate mutase	**PGlyM-2**	62.15	61.18	5.78	5.39	16.3
**C-compound and carbohydrate metabolism**								
191	AAC26045	Aconitase-iron regulated protein 1	**ACO**	102.81	98.09	5.96	5.95	14.2
325	CAN60522	Transketolase^**(*d*)**^	**TK-1**	74.77	73.77	5.97	6.36	15.3
327	CAN60522	Transketolase^**(*d*)**^	**TK-2**	74.34	73.77	6.03	6.36	10.1
378	P51615	NADP-dependent malic enzyme	**NADP-ME**	66.00	65.23	6.10	6.09	20.6
412	AAB47171	Vacuolar invertase 1	**GIN1-1**	59.07	71.55	4.27	4.60	7.9
413	CAN69570	Putative oxalyl-CoA decarboxylase^**(*d*)**^	**OxD**	60.17	61.06	5.98	5.94	23.8
431	AAB47171	Vacuolar invertase 1	**GIN1-2**	59.20	71.55	4.33	4.60	8.1
851	P52904	Pyruvate dehydrogenase E1 component subunit β, mitochondrial precursor	**PDHE1**	39.59	38.79	5.17	5.88	7.2
1109	CAN78176	Xyloglucan endotransglycosylase^**(*d*)**^	**XET**	31.65	33.18	6.19	5.98	12.5
**Photosynthesis**								
1088	CAN61828	Manganese-stablising protein/photosystem II polypeptide^**(*d*)**^	**MnSpPSII**	31.91	33.23	5.39	5.87	12.2
**Nucleobase metabolism**								
844	AAU14832	Adenosine kinase isoform 1S	**ADK**	40.04	37.44	5.60	5.07	19.7
**Amino acid metabolism**								
172	CAN63089	Glycine cleavage system P-protein^**(*d*)**^	**GCPp**	109.41	112.81	6.37	6.99	4.9
270	CAN73135	Cobalamin-independent methionine synthase^**(*d*)**^	**MetSy-1**	83.47	81.64	5.97	6.19	11.9
273	CAN73135	Cobalamin-independent methionine synthase^**(*d*)**^	**MetSy-2**	82.38	81.64	5.98	6.19	14.4
572	NP_193129	Serine hydroxymethyltransferase 4	**SHM4**	52.88	51.72	7.27	6.80	12.3
612	AAO92257	γ-aminobutyrate transaminase subunit precursor isozyme 3	**ATpL3**	50.98	57.24	6.65	6.72	20.6
654	AAG09278	Ornithine aminotransferase	**OAT**	48.56	51.32	6.21	6.44	9.4
815	P37833	Aspartate aminotransferase cytoplasmic	**AsAT**	41.43	44.51	7.31	7.75	17.7
**Transcription**								
1189	BAF46352	α chain of nascent polypeptide associated complex	**PAC**	28.78	21.92	4.06	4.32	33.7
1511	ABE01085	BTF3	**BTF3**	17.26	17.34	5.52	6.32	11.9
**Protein synthesis**								
1606	AAL13082	Putative glycine-rich RNA-binding protein	**GlyRp**	13.56	17.33	5.33	7.84	30.3
**Protein destination**								
442	Q43116	Protein disulfide-isomerase precursor	**PDIpr**	58.22	55.56	4.92	4.95	29.7
490	CAN68309	Heat shock chaperonin-binding motif^**(*d*)**^	**HSC**	56.04	41.04	4.94	4.94	17.1
1449	CAN60868	Molecular chaperone^**(*d*)**^	**MChap-1**	19.64	18.23	6.59	6.78	6.9
1513	CAN65631	Molecular chaperone^**(*d*)**^	**MChap-2**	17.26	18.15	5.73	6.17	8.8
1533	P27880	18.2 kDa class I heat shock protein	**Hsp18.2**	16.56	18.17	6.85	5.81	12.0
**Cellular communication/signal transduction**								
1016	CAN81470	Annexin^**(*d*)**^	**Annex**	34.86	35.19	6.92	7.13	29.4
**Secondary metabolism**								
986	CAN60921	Kynurenine formamidase^**(*d*)**^	**KF**	35.46	29.87	5.54	5.15	9.6
1008	CAI56335	Isoflavone reductase-like protein 6	**IFRL6**	35.23	33.93	6.09	6.02	30.8
1028	CAI56334	Isoflavone reductase-like protein 5	**IFRL5**	34.38	33.89	6.21	5.76	25.5
**Stress**								
362	NP_001031620	Binding – stress inducible protein^**(*d*)**^	**BSP**	68.17	63.71	6.05	6.00	14.9
521	AAL83720	Catalase	**CAT**	54.44	56.98	7.10	6.71	13.0
810	AAB41022	Polyphenol oxidase	**PPO-1**	41.26	67.39	6.88	6.39	8.4
819	AAB41022	Polyphenol oxidase	**PPO-2**	40.50	67.39	6.64	6.39	15.0
826	AAB41022	Polyphenol oxidase	**PPO-3**	41.09	67.39	6.81	6.39	6.1
843	AAB41022	Polyphenol oxidase	**PPO-4**	39.96	67.39	6.43	6.39	17.5
876	AAB41022	Polyphenol oxidase	**PPO-5**	38.98	67.39	5.99	6.39	9.6
906	CAN78553	Late embryogenesis abundant protein^**(*d*)**^	**LEA**	37.71	34.94	4.43	4.67	22.4
1071	CAB60154	1,3 β glucanase	**Glucβ-1**	32.26	13.37	5.99	6.11	39.3
1075	CAB91554	1,3 β glucanase	**Glucβ-2**	32.65	37.46	6.44	9.45	15.6
1148	AAQ10093	Class IV chitinase	**Chit4-1**	30.19	27.53	4.57	5.38	9.1
1177	AAB65776	Class IV endochitinase	**EnChit4**	28.50	27.24	4.93	5.38	21.1
1226	AAQ10093	Class IV chitinase	**Chit4-2**	27.66	27.53	6.87	5.38	14.4
1240	AAQ10093	Class IV chitinase	**Chit4-3**	26.99	27.53	7.35	5.38	14.4
1316	AAB61590	VVTL1	**TLP**	24.62	23.97	4.69	5.09	9.0
1318	ABC86744	Abscisic stress ripening protein	**ASR-1**	24.30	16.69	5.81	5.68	30.2
1358	ABC86744	Abscisic stress ripening protein	**ASR-2**	23.94	16.69	5.77	5.68	30.2
1385	ABB02395	Temperature-induced lipocalin	**TInLi**	22.87	21.54	6.42	6.63	13.0
1408	AAQ03092	Glutathione peroxidase	**GPOX**	21.53	18.53	6.52	6.13	23.8
1417	ABC86744	Abscisic stress ripening protein	**ASR-3**	21.22	16.69	5.73	5.68	26.2
1444	CAC16165	Pathogenesis-related protein 10	**PR10-1**	19.76	17.13	6.11	5.96	22.8
1481	AAB41022	Polyphenol oxidase	**PPO-6**	18.50	67.39	4.91	6.39	5.9
1482	AAB41022	Polyphenol oxidase	**PPO-7**	18.41	67.39	4.99	6.39	10.7
1508	CAN83049	Pathogenesis-related protein Bet v I family^**(*d*)**^	**PRBetv1**	17.20	17.10	5.15	5.12	17.2
1524	ABD78554	Pathogenesis-related protein 10.1	**PR10-2**	16.75	17.45	6.61	6.07	30.2
1768	AAB41022	Polyphenol oxidase	**PPO-8**	18.45	67.39	4.79	6.39	6.4
**Unclassified**								
476	CAN67811	Dihydrolipoamide dehydrogenase^**(*d*)**^	**Uncla-1**	56.94	49.57	6.13	7.18	9.6
1181	CAN64479	14-3-3 protein^**(*d*)**^	**Uncla-2**	28.29	28.78	4.67	4.78	16.1
1441	ABK64186	CBS domain-containing protein	**Uncla-3**	19.84	22.25	6.95	9.24	25.2
**Unknown**								
1083	NP_001061484	Protein of unknown function DUF52 family^**(*d*)**^	**Unk**	32.53	33.55	6.22	6.11	16.4

^***a***^: Experimental molecular weight (kDa) or isoelectric point

^***b***^: Theoretical molecular weight (kDa) or isoelectric point.

^***c***^: amino acid coverage (%)

^***d***^: The protein was reported as a hypothetical protein. In the features, the similarity and function of the identified genes has been annotated by the authors according to Gene Ontology .

Most of the observed variations are related to response to biotic or abiotic stresses (38%), glycolysis and gluconeogenesis (13%), C-compound and carbohydrate metabolism (13%) and amino acid metabolism (10%). The proportion of proteins involved in stress responses was quite high if compared to the functional distributions previously observed in the proteome of whole berries and isolated mesocarp, in which these proteins ranged from 8% to 19% of the identified spots [[25] and [23], respectively]. These results paralleled a recent large-scale mRNA expression analysis on the three main berry tissues [17] as well as the skin proteome analysis of cultivar Cabernet Sauvignon where most of the proteins over-expressed at maturity were involved in pathogen response [24]. This massive expression of proteins involved in stress responses may be essential to the protective function of the skin as a physical barrier between the environment and the inner tissues.

Although it is known that the biosynthesis of anthocyanins and the transcription of related genes are induced at *véraison *[9], no proteins related to this pathway were found. This failure could be ascribed to the experimental conditions used in this work. In fact, in a very preliminary analysis conducted on different genotypes using a narrower pH range (4–7) we found some really low expressed spots that were referable to enzymes involved in anthocyanin synthesis (data not shown). Nevertheless, Robinson and Davies reported that enzymes involved in this pathway are present at low levels making their assay difficult [13].

### Pathogenesis-related proteins

**Figure 5:**
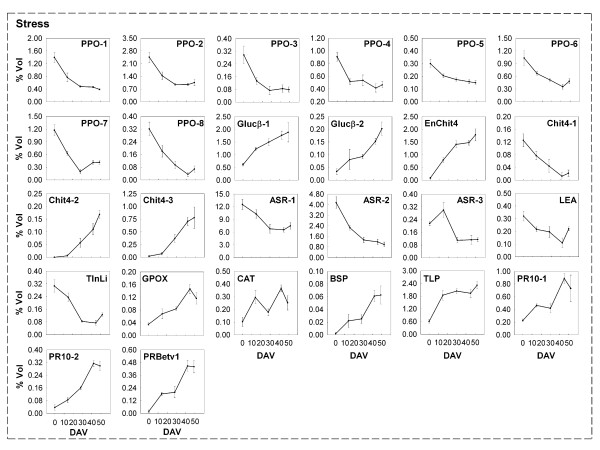
**Changes in the expression of proteins involved in stress response**. Changes in the relative spot volumes of the proteins (Table 1) involved in stress responses during five different ripening stages from *véraison *until full ripening of cultivar Barbera grape berry skins. The *véraison *stage (0 DAV) was considered as the moment when 50% of the berries started to change colour. Proteins were grouped according to their functions. Values are the mean ± SE of six 2-DE gels derived from two independent biological samples analyzed in triplicate.

Pathogenesis-related (PR) proteins belonging to class IV chitinases (Chit4), β-1,3-glucanases (Glucβ) and thaumatin-like protein (TLP) were found (Table 1 and Figure 3). PR proteins matched to a group of spots, generally low expressed at *véraison*, whose abundance abruptly rose up to the point of representing about the 20% of the total spot volume in the protein profile of ripe berries (Figure 5). Both chitinase and β-1,3-glucanase are known to have antifungal activity and presumably hydrolyse the cell walls of fungal hyphae [38,39]. In agreement with the previous proteomic studies on grape ripening [24,25], two spots were identified corresponding to β-1,3-glucanase (spots 1071 and 1075) which accumulated after *véraison*. However, data regarding the behaviour of this enzyme during berry ripening are contradictory. More than one study [38,40] pointed out that, beside the surge of chitinase activity during ripening, no β-1,3-glucanase activity was detected in grape at any stages of berry development while it was reported that the gene is expressed [37]. In spite of this, Deytieux and co-workers [24] associated the assay of the enzyme activity to the proteomic profile and found that, even if weakly correlated, both the expression and the activity of β-1,3-glucanase increased during ripening.

In addition to their involvement in osmotic stress, a role in defence against fungi in grape berries has also been suggested for thaumatin-like proteins [38]. It is interesting to observe that several studies provide evidence of the fact that chitinases and thaumatin-like proteins accumulate during berry ripening even in the absence of pathogen infections [24,38-40]. According to these results, we observed a sharp increase of these proteins moving from *véraison *to full maturation, suggesting that their expression may be developmentally regulated (Figure 5).

### Oxidative stress-related proteins

It has been proposed that the oxidative stress may play a developmental role in the ripening process [41-43]. As far as it concerns grape, this hypothesis is still a matter of debate. The data regarding the expression and the activities of proteins involved in ROS detoxification still remain unclear [25,33,37,44]. Recently, Pilati and co-workers [18] found that an oxidative burst occurs at *véraison *and that this event may modulate the expression of a gene set. Nevertheless, among the differentially expressed proteins during ripening, we identified some enzymes that are known to be involved in the oxidative stress response (*e.g*. PPO, polyphenol oxidase; GPOX, glutathione peroxidase; CAT, catalase; TInLi, temperature-induced lipocalin, Figures 3 and 5).

Polyphenol oxidases catalyze the formation of *o*-quinones, molecules involved in browning reactions as a consequence of pathogen infection, wounding and organ senescence, through the O_2_-dependent oxidation of monophenols and *o*-diphenols [45]. In addition to the described defensive role, these ubiquitous enzymes may contribute to the biosynthetic pathways leading to proanthocyanidin [46] and aurone [47]. In our work we identified 8 spots corresponding to PPO, whose expression was high at *véraison *and dropped during ripening (Table 1, Figures 3 and 5). This trend is in agreement with previous reports on this class of enzymes which are generally highly expressed and active in young developing tissues [48-51]. Dry and Robinson [52] described that the protein is synthesized as a 67 kDa precursor which is imported into the chloroplast and processed to remove a 10.6 kDa chloroplast transit peptide from the N-terminus and a 16.2 kDa peptide of unknown function from the C-terminus, thus resulting in a catalytic unit of 40.5 kDa. On the basis of the matched peptides and the deduced masses, five of the characterized proteins (spots 810, 819, 826, 843 and 876) may represent the catalytic unit. Interestingly, it is possible that spots 1481, 1482 and 1768, identified as PPO and having deduced masses of around 18 kDa, may correspond to the C-terminus of the enzyme. This was supported by the similarity of the molecular weight and by the evidence that the detected tryptic peptides are comprised in the part of the sequence between the hypothesized cleavage site and the C-terminus. This may indicate that the small terminal portion of PPO is maintained in skin cells after the cleavage from the catalytic unit. The role of this fragment is not known but it was recently indicated that its tertiary structure is likely to be similar to that of hemocyanin, an oxygen-binding protein isolated in the blood of molluscs whose main function resides in O_2_-storage and transport [53].

A spot corresponding to catalase (CAT, spot 521) presented a four-fold increase in abundance during ripening. An opposite behaviour was described for this enzyme in some recent reports on whole berries [25,40]. Although the influence of some factors can not be excluded, such as the genetic background and the environmental and seasonal conditions, these results could be explained by considering them as specific traits of the skin. For instance, it was recently discovered that the concentrations of ascorbate and glutathione in apple epidermis were 3- to 7-fold higher than in the underlying mesocarp [54]. In this view, we also observed a clear increase in the expression of a glutathione peroxidase (GPOX, spot 1408, Figure 5).

### Proteins involved in C-metabolism

Among the characterized proteins, many are involved in primary activities, such as glycolysis, gluconeogenesis, C-compounds and carbohydrate metabolism (Table 1 and Figure 3). A general picture of some traits of carbon metabolism showing the trend of these proteins is depicted in Figure 7.

**Figure 6:**
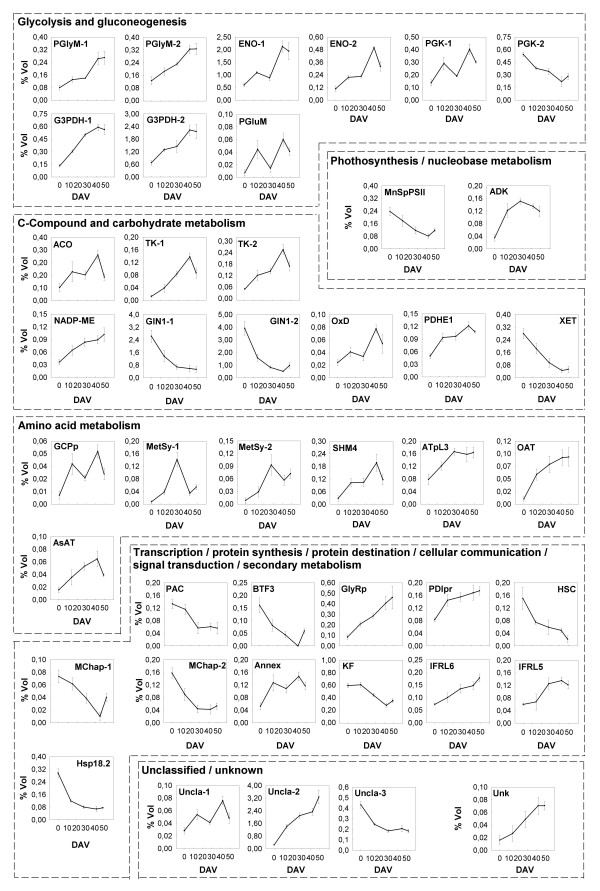
**Changes in the expression of proteins involved in C- and N-metabolism or with other functions**. Changes in the relative spot volumes of the identified proteins belonging to the indicated functional categories (Table 1), during five different ripening stages of cv. Barbera grape berry skins from *véraison *until full ripening. The *véraison *stage (0 DAV) was considered as the moment when 50% of the berries started to change colour. Proteins were grouped according to their functions. Values are the mean ± SE of six 2-DE gels derived from two independent biological samples analyzed in triplicate.

The understanding of grape assimilate partitioning, *i.e*. the process which determines the way carbohydrates are transported to the berry and how they are allocated, significantly improved in recent years. Sucrose is the preferred sugar for long-distance transfer in this species and is produced through photosynthesis in the mesophyll of mature leaves and conveyed to the berry from the phloem [55]. Until *véraison *most of the sugar imported into the berry is metabolized and so there is little storage. After *véraison*, there is an upturn in sugar levels, among which glucose and fructose, that are the most representative carbohydrates, are accumulated in roughly equal amounts in the vacuoles of the mesocarp cells [4]. A number of reports indicates that, during ripening, the localization of sucrose hydrolysis shifts from the vacuole to the apoplast [7,22,56]. This transition is associated to a decrease in the expression and activity of vacuolar invertases and a concomitant upturn of apoplastic acid invertases [7]. In agreement with these reports, we identified two spots (spots 412 and 431) corresponding to a vacuolar invertase, GIN1, showing a strong reduction in their expression after *véraison *(Figure 6).

**Figure 7:**
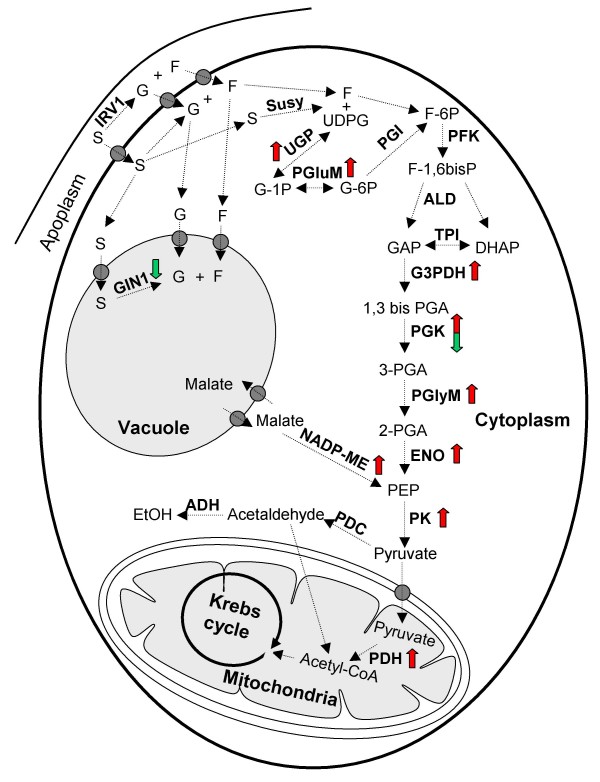
**Schematic overview of the enzymes involved in sugar and organic acid metabolisms and their connection with some intermediary activities that changed in expression in grape berry skins during five different ripe stages from *véraison *until full ripening**. The expression was evaluated by measuring relative spot volumes in the 2-DE analysis. Green or red arrows indicate whether the abundance of the identified proteins decreased or increased during ripening, respectively. IRV1, cell wall invertase, GIN1, vacuolar invertase; Susy, sucrose synthase; UGP, UDP-glucose-pyrophosphorylase; PGluM, phosphogluco-mutase; PGI, phosphogluco-isomerase; PFK, phosphofructokinase; ALD, aldolase; TPI, triosephosphate-isomerase; G3PDH, glyceraldehyde-3-phosphate-dehydrogenase; PGK, phosphoglycerate-kinase; PGlyM, phosphoglycerate-mutase; ENO, enolase; PK, pyruvate kinase; PDC, pyruvate decarboxylase; NADP-ME, NADP-dependent malic enzyme; ADH, alcohol dehydrogenase; PDH, Pyruvate dehydrogenase.

The measured drop in titratable acidity is mainly ascribed to the catabolism of malate accumulated in the vacuole during stages I and II of berry development [57]. It has been suggested that this acid is degraded in grape via at least three pathways, mainly by the cytosolic NADP-malic enzyme (NADP-ME), which catalyzes the oxidative decarboxylation of malate into pyruvate and CO_2 _[58], and, to a lesser extent, by PEP carboxykinase and the cytosolic malate dehydrogenase (cMDH) [59]. In our work we identified a spot corresponding to NADP-ME whose amount gradually increased during ripening (Figure 6). The role of this enzyme during berry development is still a matter of debate: in their tissue-specific transcriptional profile of ripe skins, Grimplet and co-workers [17] recently pointed out that the mRNA levels of several enzymes involved in malate metabolism are higher in the skins than in pulp and seeds.

In the past, several papers concerning the whole berry [25] and isolated pulp or seeds [59] reported that glycolysis is down-regulated after *véraison*. Differently, in some transcriptomic analysis conducted on the whole berry it was found that some enzymes belonging to this pathway were induced during ripening [33,40]. We have been the first, to our knowledge, who found that several glycolytic enzymes strongly increased in the skin during ripening (Figure 6). Most of them, *e.g*. phosphoglycerate mutase (PGlyM, spots 397 and 1767), enolase (ENO, spots 561 and 596), glyceraldeyde-3-phosphate dehydrogenase (G3PDH, spot 902 and 937) and phosphoglycerate kinase (PGK, spot 863), related to the energy-conserving reactions of glycolysis. These data underline the importance of distinguishing among the different berry tissues in order to understand the ripening process. In other words, the tissues could express different trends for glycolysis during ripening. In this view, we also found the concomitant high expression of NADP-ME as well as of the non-oxidative activities of the pentose phosphate pathway, such as the highly induced transketolase (TK, spots 325 and 327). These enzymes may be required in the skin for satisfying the large demand for carbon skeletons of the biosynthetic pathways operating in this tissue during ripening (*e.g*. anthocyanin synthesis).

Pyruvate may be channelled into the Krebs cycle and is converted to Acetyl-CoA by the pyruvate dehydrogenase. According to an increase in fluxes towards TCA cycle, it has been found that the subunit E1 of this enzyme (PDHE1, spot 851) is more abundantly expressed towards maturity. Aconitase (ACO) is an enzyme of the TCA and glyoxylate cycles catalyzing the reversible conversion of citrate to isocitrate. The importance of this enzyme was emphasized by Carrari and co-workers [60] who studied the *Aco-1 *tomato mutant which is characterized by a reduced expression of aconitase. Biochemical analysis of the leaves of this genotype suggested that *Aco-1 *exhibited a restricted flux through the Krebs cycle and reduced levels of Krebs cycle intermediates, with an elevated rate of photosynthesis and sucrose synthesis. The fact that *Aco-1 *leaves were also characterized by a different amino acid profile, indicates that this activity may have a role in controlling the C/N ratio and amino acid biosynthesis. We observed a spot corresponding to ACO (spot 191) whose expression sharply increased during ripening (Figure 6) as previously reported for cv. Cabernet Sauvignon skins [24] and, at the transcriptomic level, for citrus fruit flesh [61].

Oxalyl-CoA decarboxylase (OxD, spot 413) is another protein whose levels increased during ripening. This enzyme catalyses the irreversible decarboxylation of Oxalyl-CoA, derived from glyoxylic acid, to produce formyl-CoA. This activity has already been associated to grape skin during ripening [24], but further analyses are required in order to clarify its role in this process, as far as that of one- and two-carbon compounds.

### Proteins involved in N-metabolism

It has been observed that the amino acid content of the berry rises significantly during maturation and that the relative amount of different amino acids changes, with proline and arginine generally being predominant [62]. Stines and co-workers [63] suggested that proline accumulation may be achieved *via *the ornithine pathway under the control of ornithine aminotransferase (OAT), which constitutes a bridge between proline and arginine metabolism. In support of this view, we identified a very low abundance spot (< 0.1 %Vol) corresponding to OAT (spot 654) which sharply increased in expression during ripening (Figure 6).

As previously described by Giribaldi and co-workers [25], in our study we found the protein cobalamin-independent methionine synthase (MetSy, spots 270 and 273), which catalyzes the final step of methionine biosynthesis. The exact role of this enzyme, whose expression peaked in the middle of ripening, still remains unclear.

Interestingly, we identified a spot corresponding to a subunit precursor of the enzyme γ-aminobutyrate transaminase (ATpL3, spot 612) which is involved in the shunt of the aminoacid γ-aminobutyrate (GABA). To our knowledge there is no evidence of the involvement of this enzyme in the maturation of the grape berry, but it is known that it is involved in the ripening of other non-climacteric fruits, such as citrus [61,64]. According to the hypothesis proposed for citrus fruit, the GABA shunt may be active, among other things, in the regulation of cytoplasmic pH, due to the H^+^-consuming decarboxylation of glutamate, during the period of late development and ripening following citrate release from the vacuole [61].

### Other proteins

The most abundant protein found in the present work belongs to the family of ABA stress responsive elements (ASR, ca. 13% of the total volume at the first stage and ca. 6% thereafter). According to previous results, the spots 1318, 1358 and 1417 (Figure 3), which are referable to ASR, showed a downward trend during ripening (Figure 5). ASR are known to be involved in abiotic stress and fruit ripening, even though their exact role is still elusive [65,66]. The effective function has been questioned because of their very high expression level, the fact that the observed molecular masses were higher than the predicted theoretical values by a range of about 5–10 kDa and because they were found mainly in the cell wall enriched fraction [23,67].

Heat shock proteins (HSP) are usually involved in stabilizing protein folding in response to different kinds of stimuli. We identified three spots corresponding to chaperones of a predicted mass of around 18 kDa (MChap and Hsp18.2, spots 1449, 1513 and 1533, Figure 3) and a heat shock chaperonin binding motif protein (HSC, spot 490) whose levels decreased after *véraison *(Figure 6). This evidence reinforces the conclusions of da Silva and co-workers [47] who supposed that the peak of several HSPs expression at *véraison*, followed by their sudden drop, could be linked to the intense redirection of metabolism that is necessary to stabilize old and newly synthesized proteins.

Finally, some proteins characterized in this study were involved in transcription (spots 1189 and 1511), protein synthesis (spot 1606), signal transduction (spot 1016) and secondary metabolism (spots 986, 1008 and 1028). Further work is necessary to define the effective role of these proteins in the skin during ripening.

## Conclusion

This work gives new insights to the skin proteome evolution during ripening, focusing on some interesting traits of this tissue. In this view, we observed the ripening-related induction of the enzymes of the last five steps of glycolysis, although they had been described as down-regulated in previous studies performed on whole fruit. These variations were accompanied by the rise of the levels of other important proteins of primary metabolism, such as malic enzyme, aconitase, pyruvate dehydrogenase and transketolase.

These results paved the way for investigations on the role of this tissue that has to respond to specific metabolic requests being the site of important biosynthetic pathway (*e.g*. anthocyanin). Moreover, the data emphasize the relevance of the skin as a physical barrier playing an important role in berry protection. In fact, the levels of many proteins known to take part in (a)biotic stress responses vary during the five analyzed stages. Many of them (*i.e*. chitinase, thaumatin-like, abscissic stress ripening protein, polyphenol oxidase) are the most expressed proteins found in this work and are characterized by the most abrupt variations in accordance to their possible developmental regulation.

## Methods

### Plant material and experimental design

Experimental material was harvested during the 2005 growing season from *Vitis vinifera *L. cv. Barbera grapevines, grown at the Experimental Station of the Ente Regionale per i Servizi all'Agricoltura e alle Foreste (E.R.S.A.F.) of Regione Lombardia (Pavia, Italy).

Samples were collected at five different ripening stages from *véraison*, until full ripening (corresponding to 58, 72, 86, 100, 107 days after blooming). We considered the *véraison *stage as the moment when 50% of the berries started to change colour.

Two hundred berries were collected at each sampling date. Berries were equally sampled on a single cluster per plant across 20 plants. Immediately after harvest, the skins were collected by squishing the berries in order to remove seeds and the bulk of the mesocarp, then pressing and smearing the inner part of the skin on two layers of cheesecloth to completely take away the residual pulp. Skins samples were split into two technical replicates. The samples were frozen in liquid nitrogen and stored at -80°C until use. Each technical replicates was subjected to independent protein extraction. Three gels were run for each extraction. At all stages, samples of whole fresh berries, obtained as described above, were immediately used to measure total soluble solids, pH and titratable acidity.

### Determination of physiological parameters

In order to assess the progress of grape berry ripening and to associate the physiological phases to the observed changes in protein expression, total solids, pH, titratable acidity and anthocyanins were evaluated on five stages of ripening, starting from *véraison *to full maturation.

Total soluble solids (°BRIX), pH and titratable acidity were measured in grape juice, obtained by pressing fresh berries with a small hand-crank press, using a hand held refractometer (ATAGO CO., Ltd), a pH meter (Hanna HI 221) and an automatic titrator (Crison Compact Titrator) titrating in the presence of NaOH. Anthocyanins were extracted from the skins as previously described by Fumagalli and co-workers [68]. The anthocyanins concentration was evaluated by measuring the absorbance of the extract at a wavelength of 535 nm and referring the values to a malvidin-3-glucoside calibration curve.

**Figure 8:**
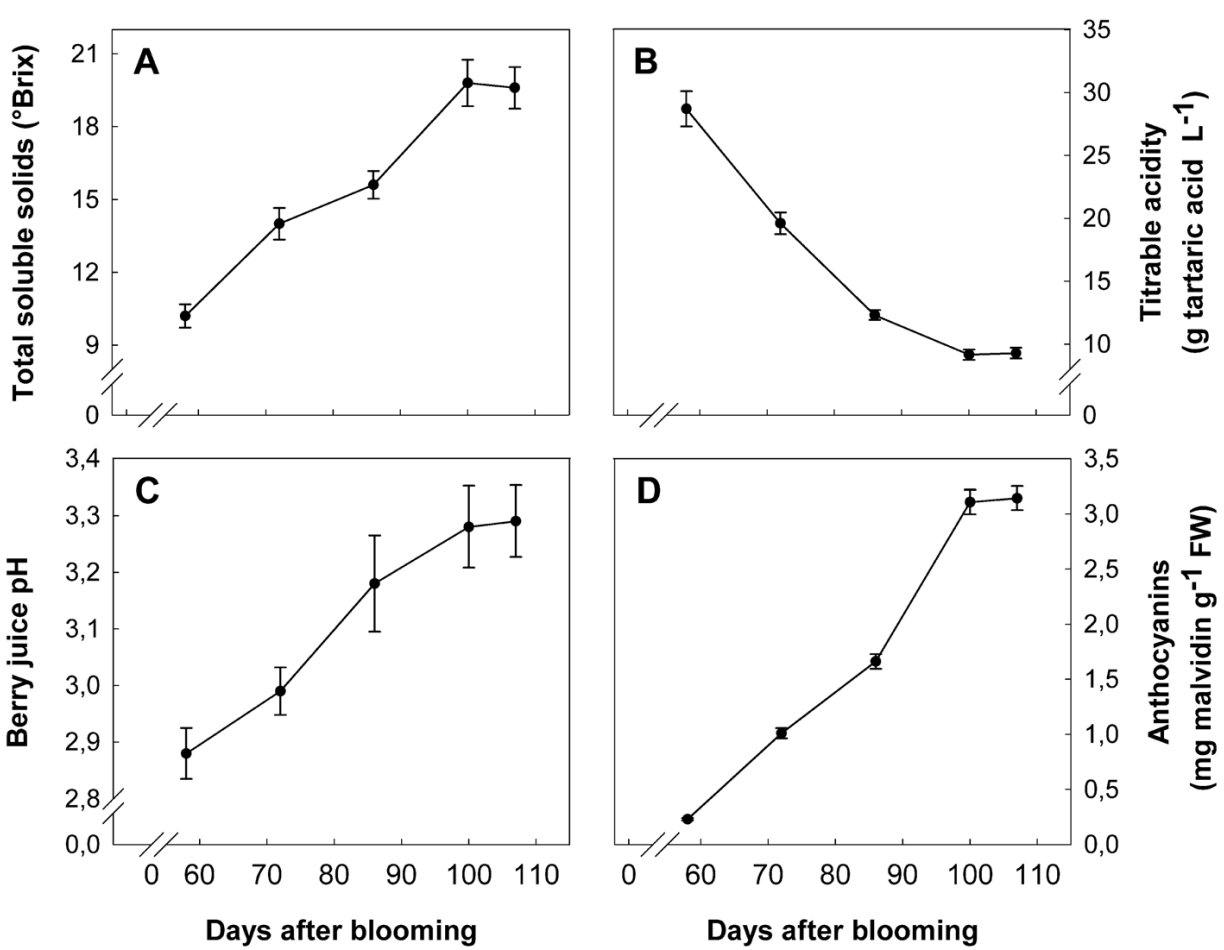
**Biochemical changes occurring during the ripening of Barbera berries**. Changes in the physiological parameters were measured during five different ripening stages of cultivar Barbera grape berries from *véraison *until full ripening. The *véraison *stage (58 days after blooming) was considered as the moment when 50% of the berries started to change colour. A, total soluble solids; B, titratable acidity; C, berry juice pH; D, total anthocyanin contents. The data are the means ± SE of three experiments run in triplicate (n = 9).

Considering the whole period, a sharp increase in the anthocyanin content of the skin, soluble solids and pH in berry juice was measured, while a reduction in titratable acidity occurred at the same time (Figure 8). In detail, we observed a 10-fold surge in the anthocyanin level and a 2-fold upturn of soluble solids, accompanied by a pH shift of 0.4 and a 3-fold decrease in titratable acidity. The rate of sugars and anthocyanins accumulation as well as the changes in pH and titratable acidity were almost constant until the fourth stage, while no significant variations for these parameters were observed between the fourth and the fifth sampling stages.

### Protein extraction and quantification

Frozen samples (5 g) were finely powdered in liquid nitrogen using a pestle and mortar, homogenized with cold (-20°C) acetone, washed twice on Whatman 41 filter paper (Whatman International Ltd) with cold acetone and finally dried under vacuum. The acetone powder was then resuspended in 20 mL of extraction buffer [0.7 M sucrose, 0.5 M Tris-HCl pH 8, 10 mM disodium EDTA salt, 4 mM ascorbic acid, 1 mM PMSF, 1 μM leupeptin, 0.1 mg mL^-1 ^Pefabloc (Fluka), 0.4% (v/v) β-mercaptoethanol] on ice, incubated in a 4°C cold room under shaking for 30 min and then centrifuged at 13000 *g *for 30 min. The resultant supernatant was extracted as previously described by Hurkman and Tanaka [32] by the addition of an equal volume of ice-cold Tris-buffered phenol (pH 8). The sample was shaken for 30 min at 4°C, incubated for 2 h at 4°C and finally centrifuged at 5000 *g *for 20 min at 4°C to separate the phases. The upper phenol phase was collected, while the aqueous phase at the bottom was back-extracted with an equal volume of phenol. Proteins were precipitated by the addition of five volumes of ice-cold 0.1 M ammonium acetate in methanol to the phenol phase, then vortexed briefly and finally incubated at -20°C overnight. Precipitated proteins were recovered by centrifuging at 13000 *g *for 30 min, then washed again with cold methanolic ammonium acetate and three times with cold 80% (v/v) acetone. The final pellet was dried under vacuum and dissolved in IEF buffer [7 M urea, 2 M thiourea, 3% (w/v) CHAPS, 1% (v/v) NP-40, 50 mg mL^-1 ^DTT and 2% (v/v) IPG Buffer pH 3–10 (GE Healthcare)] by vortexing and incubating for 1 h at room temperature. The sample was centrifuged at 10000 *g *for 10 min and the supernatant stored at -80°C until further use. The protein concentration was determined by 2-D Quant Kit (GE Healthcare).

### 2-DE

The protein sample (200 μg) was loaded on pH 3–10, 24 cm IPG strips passively rehydrated overnight in 7 M urea, 2 M thiourea, 3% (w/v) CHAPS, 1% (v/v) NP-40, 10 mg mL^-1 ^DTT and 0.5% (v/v) IPG Buffer pH 3–10. IEF was performed at 20°C with current limit of 50 μA/strip for about 90 kVh in an Ettan IPGphor (GE Healthcare) using the following settings: 5 min gradient 200 V, 1 h at 200 V, 5 min gradient 500 V, 1 h at 500 V, 5 min gradient 1000 V, 6 h at 1000 V, 3 h gradient 8000 V and 9 h at 8000 V. After IEF, strips were equilibrated by gentle shaking for 15 min in an equilibration buffer [100 mM Tris-HCl pH 6.8, 7 M urea, 2 M thiourea, 30% (w/v) glycerol, 2% (w/v) SDS] added with 0.5% (w/v) DTT for disulfide bridges reduction and for an additional 15 min in the same equilibration buffer to which was added 0.002% (w/v) bromophenol blue and 4.5% w/v iodoacetamide for cysteine alkylation. Second-dimensional SDS-PAGE [69] was run in 12.5% acrylamide gels using the ETTAN DALT *six *apparatus (GE Healthcare). Running was first conducted at 5 W/gel for 30 min followed by 15 W/gel until the bromophenol blue line ran off.

### Protein visualization and image and data analysis

Proteins were stained using the colloidal Coomassie Brilliant Blue G-250 (cCBB) procedure, as previously described by Neuhoff and co-workers [70]. The gels were scanned in an Epson Expression 1680 Pro Scanner and analyzed with ImageMaster 2-D Platinum Software (GE Healthcare). Automatic matching was complemented by manual matching. Molecular weights of the spots were deduced on the basis of the migration of SigmaMarkers™ wide range (MW 6.500 – 205.000), while p*I *was determined according to the strip manufacturer's instructions (GE Healthcare).

Relative spot volumes of the six replicate gels of the five ripening stages were compared and were analyzed according to the ANOVA test to verify whether the changes were statistically significant (*p *< 0.01). Only spots showing at least a two-fold change in their relative volumes were considered for successive analysis. Significant differences were analyzed through the two-way hierarchical clustering methodology using the software PermutMatrix [71,72]. For this purpose, the data produced by the analysis of 2-DE gels were converted into a binary matrix replacing the missing values by zero. The row by row normalization of data was performed using the classical zero-mean and unit-standard deviation technique. Pearson's distance and Ward's algorithm were used for the analysis.

### In-gel digestion, mass spectrometry and protein characterization

Spots were excised from cCBB-stained 2-DE gels and in-gel digested as previously described by Magni and co-workers [73]. The extracted tryptic fragments were resuspended in 0.1% (v/v) formic acid and analysed by LC-ESI-MS/MS. For all the experiments a Finnigan LCQ Deca XP MAX IT mass spectrometer equipped with a Finnigan Surveyor (MS Pump Plus) HPLC system (Thermo Electron Corporation) was used. Chromatography separations were conducted on a BioBasic C18 column (180 μm I.D. × 150 mm length and 5 μm particle size), using a linear gradient from 5% to 80% solvent B [solvent A: 0.05% (v/v) formic acid; solvent B: ACN containing 0.05% (v/v) formic acid] with a flow of 2.5 μL/min. The capillary temperature and the spray voltage were set at 220°C and at 3.0 kV, respectively. For MS/MS scans the normalized collision energy was set at 35%. Acquisitions were performed in data-dependent MS/MS scanning mode and enabling a dynamic exclusion window of 3 min.

Protein identifications were conducted by using TurboSEQUEST^® ^incorporated in BioworksBrowser 3.2 (Thermo Electron Corporation) by correlation of uninterpreted spectra to the entries of NCBI NR non-redundant (*i*), *Vitis *protein subset (*ii*) and *Vitis *EST subset (*iii*) databases extracted from the NCBI NR non-redundant database (*ii*) and ESTdb others (*iii*), downloaded from the National Center for Biotechnology Information (NCBI). The software was set to allow two missed cleavages per peptide and to take into account fixed modification of cysteine carboxyamidomethylation and variable modification of methionine oxidation. The parent ion and fragment ion mass tolerance were set to ± 2 Da and ± 1 Da, respectively. In order to identify proteins, only peptides with Xcorr ≥ 1.5 (+1 charge state), ≥ 2.0 (+2 charge state), ≥ 2.5 (≥ 3 charge state), peptide probability < 1 × 10^-3^, ΔCn ≥ 0.1 and Sf ≥ 0.70 were considered. Regarding protein identification by sequence similarity search, identified peptides were aligned against the NCBI NR non-redundant database using the FASTS algorithm [74]. Theoretical molecular weight and p*I *of characterized proteins were calculated by processing sequence entries at . Protein functions were assigned to MIPS FunCat  according to their role described in the literature.

## Abbreviations

NP-40 octylphenoxy polyethoxy ethanol; cCBB Colloidal Coomassie Brilliant Blue.

## Authors' contributions

ASN carried out protein extraction, 2-DE, gel analysis, clustering and statistical analysis and wrote the initial manuscript draft. BP contributed to the conception of the experimental design, carried out protein characterization by LC-ESI-MS/MS, analyzed the MS data, participated in writing the methods section of the manuscript. MR performed metabolite analyses. OF and AS participated to the manuscript revision. MC contributed to the interpretation of the results and took part to the critical revision of the manuscript. LE conceived the study, coordinated the experiments, wrote and edited the manuscript. All authors read and approved the final manuscript.

## Supplementary Material

Additional file 1 Data on protein identification by LC-ESI-MS/MS and bioinformatic analysis. table shows the sequence of all the peptides identified by MS/MS fragmentation and the statistical information related to peptides, proteins and alignment analyses. Click here for file
